# Will We Be Known by Another Name?

**Published:** 1894-07

**Authors:** 


### Editorial.

### WILL WE BE KNOWN BY ANOTHER NAME?

THE changes in the use of language and the definition of terms co-exist with the evolution of thought and the variations in the development of civilizations. The word used to-day to express an idea may not be satisfactory at a future period, and it is, therefore, quite essential that ideas should be clothed in new forms of expression to correspond with the modifications made necessary by the changed experiences.

When this general thought is applied to our profession it is recognized that we have reached a period of unrest, a desire almost morbid in character to alter the expressions of the past into something more definitely fitting the reconstructed ideas prevailing at present. This is particularly noticed in the attempts being made everywhere to change the nomenclature of dentistry to something different from that heretofore recognized. That this movement will have a good effect who may doubt? but that it will result in radical changes may well be questioned, for, as was remarked in a former article, forced changes in language, whether technical or general, cannot be made by proclamation or by learned essays, but must be the gradual development through forces not always clearly explainable, but still always consistent with the necessities of professions and peoples.

Words have their time of existence; their birth, slow development into the ideas of the world, their gradual change by usage, and, finally, they cease to represent the new thought and become antiquated, and are eventually lost.

The development of dentistry has given rise to many technical terms, not always well chosen, but they exist and are in active life to-day. The old age of these has not yet arrived, but it will gradually come as the profession develops, and new forms of expression are born of necessity.

It may be well, therefore, not to attack the old in endeavors to upset the forms of the past, lest in so doing the ideas they represent may be in a measure lost, and this is always to be deprecated. Dentistry, though comparatively young, has its "lost arts," and it is feared, if the iconoclastic efforts of some prevail, these may be multiplied, to the serious detriment of professional ability.

There is, however, a danger of fostering conservatism in this direction to an extent which may prove a serious bar to progress, and while the too liberal resort to new forms on the one hand should be regarded with disfavor, on the other the dogmatic assertion that no good can come from this effort to change technical terms must be accepted as a conservative error.

This train of thought has been presented to the writer by noticing that our French co-workers have adopted a term that seems to indicate that the twentieth century will show that even the title we have been known by from time immemorial will have become obsolete. It is not a new term, but is new to dental thought, and yet it carries with it so much that it seems destined eventually to relegate the word dentist to oblivion. The word in question is Stomatology.

The French have contributed largely to the advance of scientific dentistry. To Fauchard, Jourdain, Delabarre, etc., we owe a lasting debt, and now, under the leadership of the celebrated Magitot, there has been organized "The Society of Stomatology of Paris." To this body the word dentistry seems to have become effete. It was satisfactory when the operators of a former period were content with the care of the teeth, but at this time a word is required embracing all operations of the oral cavity in all their varied presentations, and no other one expresses this so completely as that adopted.

It seems as though the dentist of the future will cease to use the name as descriptive of his work, and he must by force of development become a Stomatologist, and be allied with other specialists, such as Dermatologist, Otologist, Gynæcologist, etc.

The force of this, it is surmised, cannot be controverted. The term dentistry has ceased to represent modern thought and practice on this subject, and must, therefore, give way to the newer word, which aligns the practitioners of this science and art along with the specialists of medicine.

Whatever may be the preferences of individuals, however much the love of the old term may cling to us, it is quite evident that the trend of thought is towards a coalescing of dentistry with medicine, and the terms used must be in accordance with this evolutionary process.

It is not probable that the world at large will ever accept the change proposed, so that there is no danger of dentistry ceasing to be a part of the vocabulary of civilized peoples, but for professional use it is probably doomed, and we will be wise to recognize the fact.

That this is being made apparent in this country as a truth is evidenced by the recent organization of a society in Philadelphia, to be known as "The Academy of Stomatology." The strict scientific basis of this organization is more encouraging than even its name, for it aims to cultivate the true scientific spirit in all of its work. If this be carried out it will mark a new era in dental society life and procedures.

We must, therefore, welcome this word into the professional family, and cannot but feel that its adoption means the inauguration of a new order of things in dentistry, not the sacrifice of the cherished old, but the advancement of the entire body to meet the ever-changing current of professional thought.

